# A Deep-Learning Pipeline for TSS Coverage Imputation From Shallow Cell-Free DNA Sequencing

**DOI:** 10.3389/fmed.2021.684238

**Published:** 2021-12-03

**Authors:** Bo-Wei Han, Xu Yang, Shou-Fang Qu, Zhi-Wei Guo, Li-Min Huang, Kun Li, Guo-Jun Ouyang, Geng-Xi Cai, Wei-Wei Xiao, Rong-Tao Weng, Shun Xu, Jie Huang, Xue-Xi Yang, Ying-Song Wu

**Affiliations:** ^1^Key Laboratory of Antibody Engineering of Guangdong Higher Education Institutes, School of Laboratory Medicine and Biotechnology, Southern Medical University, Guangzhou, China; ^2^Division of in vitro Diagnostic Reagents, National Institutes for Food and Drug Control (NIFDC), Beijing, China; ^3^Guangzhou XGene Co., Ltd., Guangzhou, China; ^4^Guangzhou Darui Biotechnology Co., Ltd., Guangzhou, China; ^5^Department of Breast Surgery, The First People's Hospital of Foshan, Foshan, China; ^6^Sun Yat-sen Memorial Hospital, Sun Yat-sen University, Guangzhou, China; ^7^Department of Medical Oncology, State Key Laboratory of Oncology in South China, Collaborative Innovation Center for Cancer Medicine, Sun Yat-sen University Cancer Center, Guangzhou, China

**Keywords:** cell-free DNA, deep learning, nucleosome footprint, whole-genome sequencing, autoencoder

## Abstract

Cell-free DNA (cfDNA) serves as a footprint of the nucleosome occupancy status of transcription start sites (TSSs), and has been subject to wide development for use in noninvasive health monitoring and disease detection. However, the requirement for high sequencing depth limits its clinical use. Here, we introduce a deep-learning pipeline designed for TSS coverage profiles generated from shallow cfDNA sequencing called the Autoencoder of cfDNA TSS (AECT) coverage profile. AECT outperformed existing single-cell sequencing imputation algorithms in terms of improvements to TSS coverage accuracy and the capture of latent biological features that distinguish sex or tumor status. We built classifiers for the detection of breast and rectal cancer using AECT-imputed shallow sequencing data, and their performance was close to that achieved by high-depth sequencing, suggesting that AECT could provide a broadly applicable noninvasive screening approach with high accuracy and at a moderate cost.

## Introduction

Plasma cell-free DNA (cfDNA) is an intensively investigated biomarker that has been widely used for noninvasive cancer evaluation and prenatal testing (1–4). Because cfDNA predominantly consists of the nucleosome-protected DNA of apoptosis cells, recently, cfDNA has been proven to powerfully imply nucleosome positioning, and it can be further used to predict the status of gene expression based on the nucleosome occupancy level at transcription start sites (TSSs) (3, 5, 6). Therefore, cfDNA TSS coverage profiles are informative for biological process and regulatory networks in organisms, and a set of noninvasive cfDNA coverage-based screening methods have been developed for use in the detection of cancer (7–9), evaluation of therapeutic effects in cancer, the prediction of pregnancy complications (3, 10), health monitoring in pregnancy (11), and other uses. However, most of these methods require deep whole-genome sequencing data, which limits its routine clinical usage due to cost (7). Existing methods based on low-coverage cfDNA sequencing often suffer from the insufficient accuracy of clinical applications (3, 10, 11). Therefore, a new approach is needed to balance between the cost of cfDNA sequencing and the accuracy of TSS coverage profiles.

Computational approaches have been designed to improve the measurement of genomic or transcriptomic spectra generated from low-coverage sequencing data, particularly from single-cell RNA-seq data and single-cell ATAC-seq data (12–15). These algorithms were designed using a range of principles and models and perform well enough to impute the missing values caused by dropouts in single-cell sequencing data. However, it may not be possible to directly apply these algorithms to TSS coverage data because most were designed for sparse single-cell sequencing data, while TSS coverage data show much less sparsity. Moreover, the distribution of TSS coverage also differs from that of single-cell sequencing data, which may not be well fitted to the algorithms for single-cell data.

**Figure 1:**
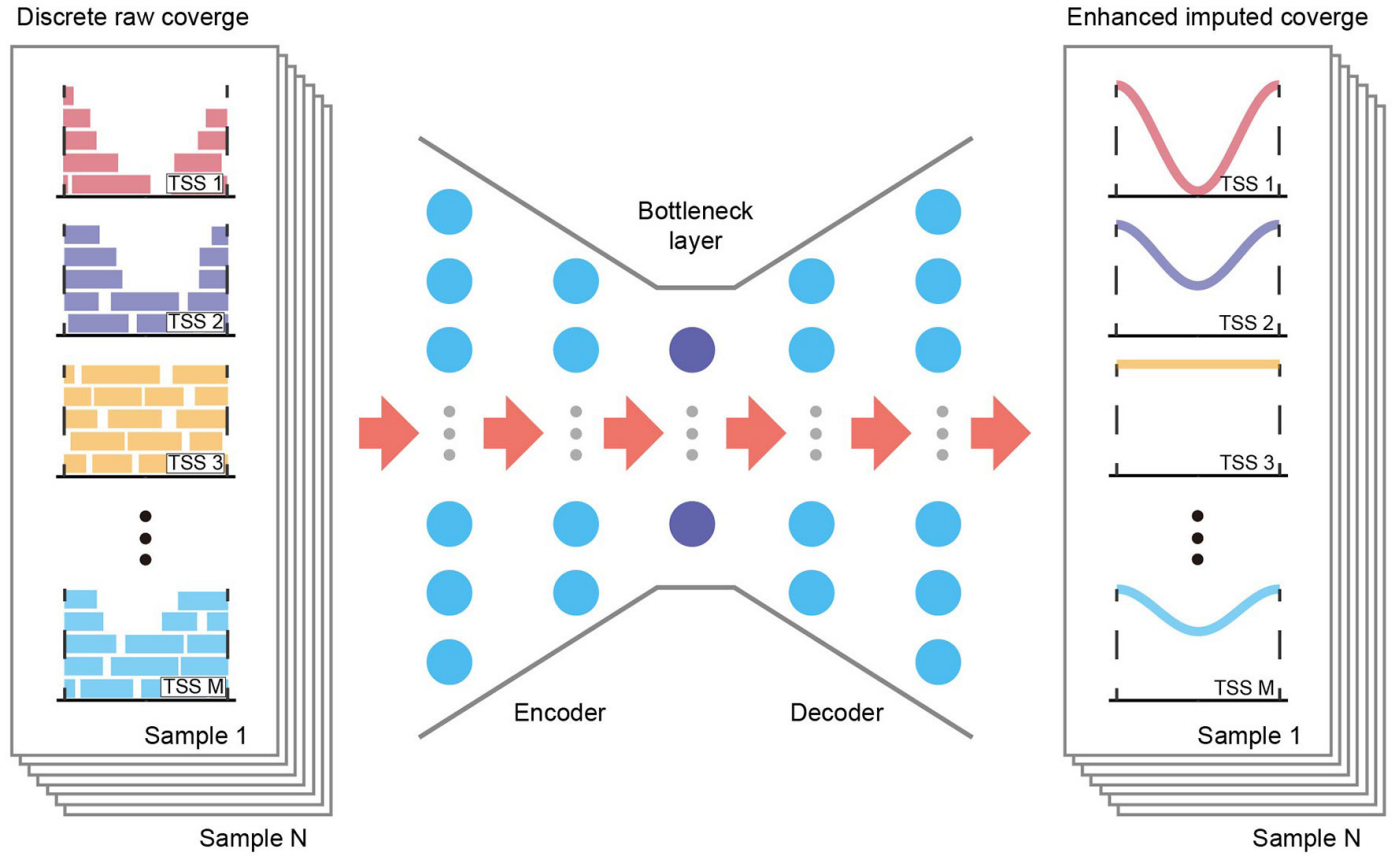
Overview of the AECT framework. AECT is an autoencoder-based framework that has an encoder and a decoder. The autoencoder is a five-layer fully connected neural network with 128, 64, 32, 64, and 128 neurons. It uses discrete raw TSS coverage generated from shallow cfDNA sequencing data as input, and it outputs imputed coverage profiles.

Although existing methods may fail to account properly for TSS coverage data, they highlight the potential to capture accurate TSS coverage profiles and extract data structures from shallow sequencing data. One of the most popular such methods, using autoencoder frameworks, may apply to TSS coverage data due to its flexibility and scalability (13, 14). An autoencoder is a deep generative model that learns the latent distribution of the input data unsupervised through a recognition model (encoder) and subsequently reconstructs the data with a generative model (decoder, Figure 1). During deep learning, an autoencoder shares information across features and thereby recovers the complexity and nonlinearity of gene–gene relationships. Adjusting the dimensions of the bottleneck layer in the neural networks forces the autoencoder to learn only the essential biological features, and it generates imputed data without the random noise introduced by low coverage.

Here, we introduce the Autoencoder of cfDNA TSS (AECT) coverage profile, a method of denoising TSS coverage profiles generated by shallow cfDNA sequencing. A set of pre-processing steps for cfDNA sequencing data, including GC bias adjustment and copy number normalization, are also integrated. The effectiveness of AECT was validated using multiple datasets. Outperforming other tools designed for single-cell sequencing data, AECT generated comparable accuracy of TSS coverage profiles as high-depth cfDNA sequencing data, and it was sufficiently powerful to uncover the latent biological features in shallow cfDNA sequencing generated from healthy donors and tumor patients. In sum, AECT greatly improves the performance of shallow sequencing-based cancer detection and sheds a light on the clinical use of cfDNA sequencing at an acceptable cost.

## Materials and Methods

### Overview of the AECT Algorithm

AECT is a deep neural network autoencoder, implemented with the Keras framework and TensorFlow in the backend. It uses TSS profiles as its input layer and predicts imputed profiles as the output layer. By default, five fully connected hidden layers with 128, 64, 32, 64, and 128 neurons are used to compress and reconstruct the data using the MSE loss function (Figure 1). The rectified linear unit (ReLU) is used as an activation function for hidden layers, and mini-batch sizes of 32 are used to train the neural network. The training stops if it reaches 500 epochs or if validation loss does not improve for 15 epochs. It is worth mentioning that the default hyperparameters work well for datasets in this study; however, there might be a better parameter combination in another dataset. Hence, we provided a set of parameters in AECT software for model tuning.

### Related Work

A few related studies review to our study. A set of deep learning-based denoising model for single cell sequencing data, including DCA (13), DeepImpute (15), and SCALE (14), inspired us for developing an autoencoder model for shallow cfDNA sequencing data. And a set of studies (3, 11, 16, 17) also provide the theoretical basis for physiology and pathology status prediction using cfDNA-based nucleosome footprint.

### Human Cancer and Normal Samples

The samples used in the study have been described in previous studies (16, 17). The first study collected plasma cfDNA sequencing data of breast cancer patients, benign breast lesion patients, and healthy donors (17). The other study collected plasma cfDNA sequencing data of rectal cancer patients (16). After discarding samples with insufficient data size and incomplete information, a total of 635 samples were used: 168 from breast cancer patients, 140 from benign breast lesion patients, 168 from rectal cancer patients, and 159 from healthy donors. Detailed sample information, including age, sex, tumor stages and subtypes, is presented in Supplementary Table 1.

### Data Preprocessing

Single-end sequencing data were generated from the Ion Proton platform (ThermoFisher Scientific, USA). After low-quality sequencing reads were removed, high-quality reads were aligned to the hg19 human reference genome using TMAP (v5.4), and PCR duplication was removed. Because AECT uses the read frequencies of each TSS region, AECT users could also use hg38 as reference genome. GC-bias correction and copy number change normalization were performed with the deepTools correctGCBias algorithm (18, 19), and DNAcopy R package, respectively. Similar to what was done previously (3), the raw read counts and GC-corrected read frequencies for each TSS region [defined as the region ranging from −1 to +1 KB around the TSS, a total of 41,784 TSSs annotated in RefSeq database were used in the analysis] were calculated using bedtools (20), and then were divided by the relative copy numbers of each region. The TSS coverage profiles for each sample were subsequently normalized using the reads per kilobase per million mapped reads method and were submitted as the input for imputation algorithms.

### Downstream Functional Analyses

To compare the coverage of TSSs between groups, *p*-values were calculated using the Wilcoxon rank-sum test and then were adjusted to the FDR, using the Holm procedure. TSSs with FDR < 0.1 were selected as altered TSSs for downstream functional analyses, and gene-set annotation and functional enrichment analysis (on GO database) were performed using Metascape with default parameters (21). To measure the gene–gene relationships raised by imputation, the correlations for TSSs were calculated using the Pearson correlation coefficient, and PCAs were used to visualize sample similarity, based on the TSS profiles.

### Prediction Model Construction and Validation

To compare cancer detection performance for TSS profiles generated by imputation algorithms, we used a penalized logistic model to select variables for model construction. The training procedure was kept consistent between raw and imputed datasets to enable comparison. The R package glmnet was used to perform the least absolute shrinkage and selection operator (LASSO), and lambda values were determined by 10-fold cross-validation. For each model, 1,000-times bootstrapping was used to test the robustness of the candidate genes chosen by the model. To reduce overfitting, we constructed the final prediction model using 100 candidate genes that were most often seen in the bootstrapping. The performance of the classifiers was evaluated on the training cohort and validation cohorts using receiver operating characteristics generated by the pROC R package (22).

### Third-Party Imputation Algorithms

For ease of comparison, we used the latest version of MAGIC (v1.5.5) (12), DCA (v0.2.3) (13), DeepImpute (v1.0.0) (15), and SCALE (v1.0.2) (14) at the default parameters.

## Results

### GC Bias Adjustment Reduces Batch Effects in TSS Coverage Profiles

GC content bias influences the number of reads that are mapped to a genomic region, confounds the quantification of TSS coverage profiles, and is a major cause of batch effects in cfDNA sequencing data (19). To address this issue, we developed a deepTools-based (18) pipeline to correct GC bias at each TSS region (±1 kbps surrounding a TSS). Noninvasive prenatal testing (NIPT) data generated from different experimental batches were selected to evaluate correction performance. As shown in Supplementary Figure 1, uncorrected NIPT data showed visible batch differences, but our GC-correcting pipeline reduced batch effects (Supplementary Figures 1A–D). Moreover, fetal fraction-enriched NIPT samples with cfDNA fragment length selection, which have different TSS coverage profiles with ordinary NIPT samples, were also added to the analyses. Using principal component analysis (PCA), GC-corrected NIPT samples were grouped according to whether they did or did not have fetal fraction enrichment (Supplementary Figures 1E–H), suggesting that our GC-correcting method reduced batch effects without over-correcting biological variation.

### AECT Improves the Accuracy of TSS Coverage Profile in Low-Coverage Data

We developed AECT to impute precise TSS coverage (Figure 1). The AECT uses a GC-adjusted TSS coverage matrix as input data, captures a latent distribution using five hidden layers with 128, 64, 32, 64, and 128 neurons, and reconstructs the data using the mean squared error (MSE) loss function. Detailed information on AECT is presented in the Methods section.

**Figure 2:**
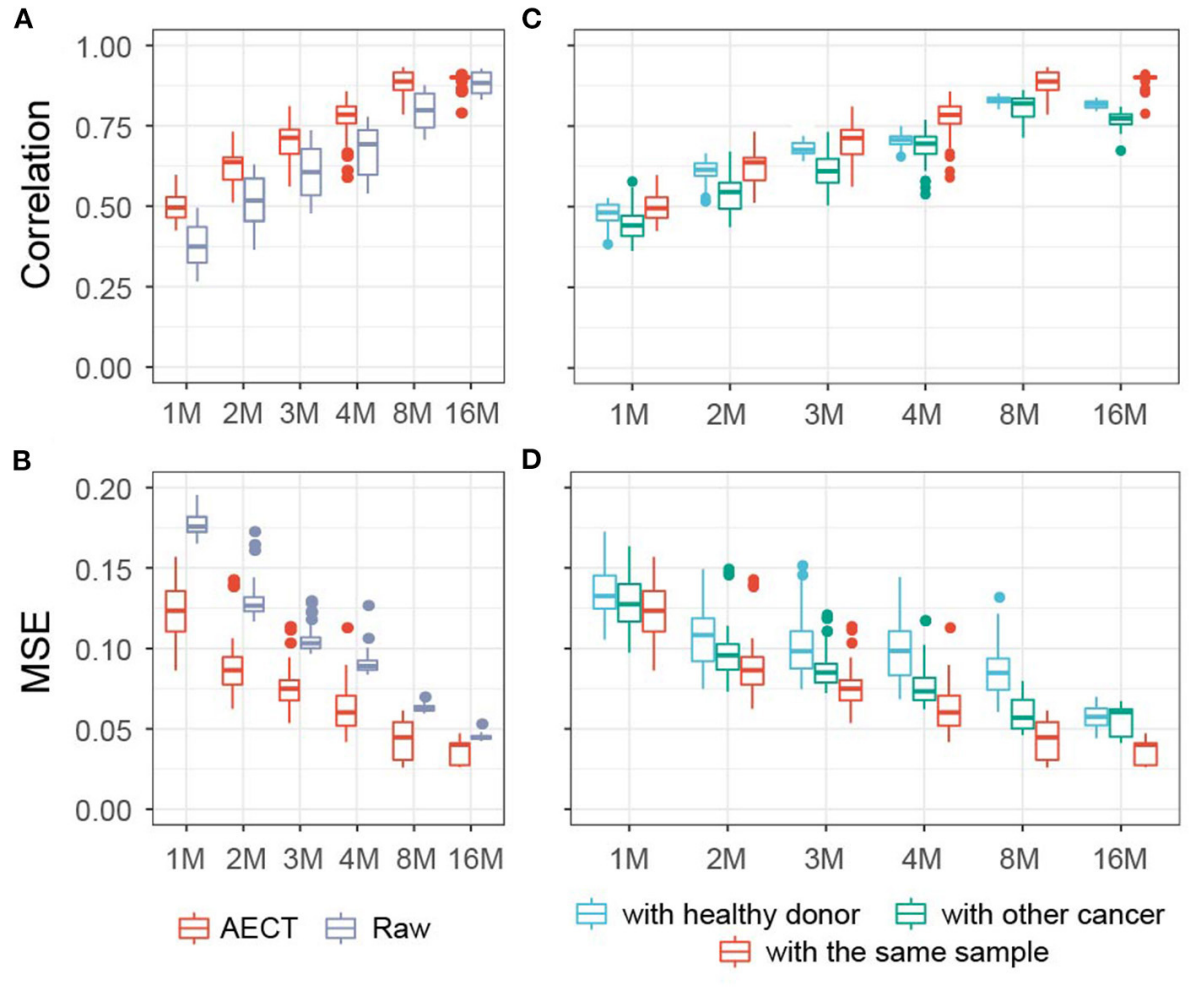
AECT improved accuracy of TSS coverage profiles in simulated data of cancer patients. **(A,B)** AECT increased the Pearson correlation coefficient **(A)** and decreased the MSE **(B)** for shallow data and original high depth data. **(C,D)** AECT generated a higher Pearson correlation coefficient **(C)** and a lower MSE **(D)** with the original high-depth data than with the high-depth data of other cancer samples and healthy donors. The x-axis represents the read counts for the simulated shallow-sequencing data. The box represents the interquartile range, the horizontal line in the box is the median, and the whiskers represent 1.5 times the interquartile range.

As a proof-of-principle and to explore the properties of our approach, we applied AECT to mimic low cfDNA-sequencing data. Five high-depth sequenced samples (>400 M reads) (6), including three healthy, one breast cancer, and one colorectal cancer tissues, were randomly down-sampled into simulated shallow sequencing data (ranging from 1 to 16 M reads, 10 samples per depth group). AECT was performed on each depth group, and the Pearson correlation coefficient and MSE were used to measure the similarities between AECT-imputed shallow data and the original high-depth data. As expected, AECT significantly increased the correlation coefficient and reduced the MSE of cancer patients (Figures 2A,B) and healthy donors (Supplementary Figures 2A,B). Even for simulated data with only 1 M reads, it increased the accuracy (Figures 2A,B; Supplementary Figures 2A,B). AECT-imputed data presented higher similarity to their original sources (Figures 2C,D; Supplementary Figures 2C,D) than other high-depth samples. It is worth noting that shallow data from healthy donors showed higher similarity with other healthy donors than with high-depth cancer samples, which implies that our AECT model captured not only the features of each sample but also the biological characters underlying them.

We also used four representative algorithms designed for single-cell sequencing imputation, namely, MAGIC (12), DeepImpute (15), DCA (13), and SCALE (14), on shallow data (Supplementary Figures 3, 4). Of the four algorithms, MAGIC and SCALE obtained higher correlations and lower MSEs than AECT; however, these two could not distinguish sample type or sample resource. The other two algorithms, DCA and DeepImpute, were comparable to AECT, but they also failed to identify the origin of shallow data for some samples. In summary, AECT yielded higher similarity to original high-depth data and effectively discriminated the sample origins.

### AECT Captures Latent Features in Healthy Donors

**Figure 3:**
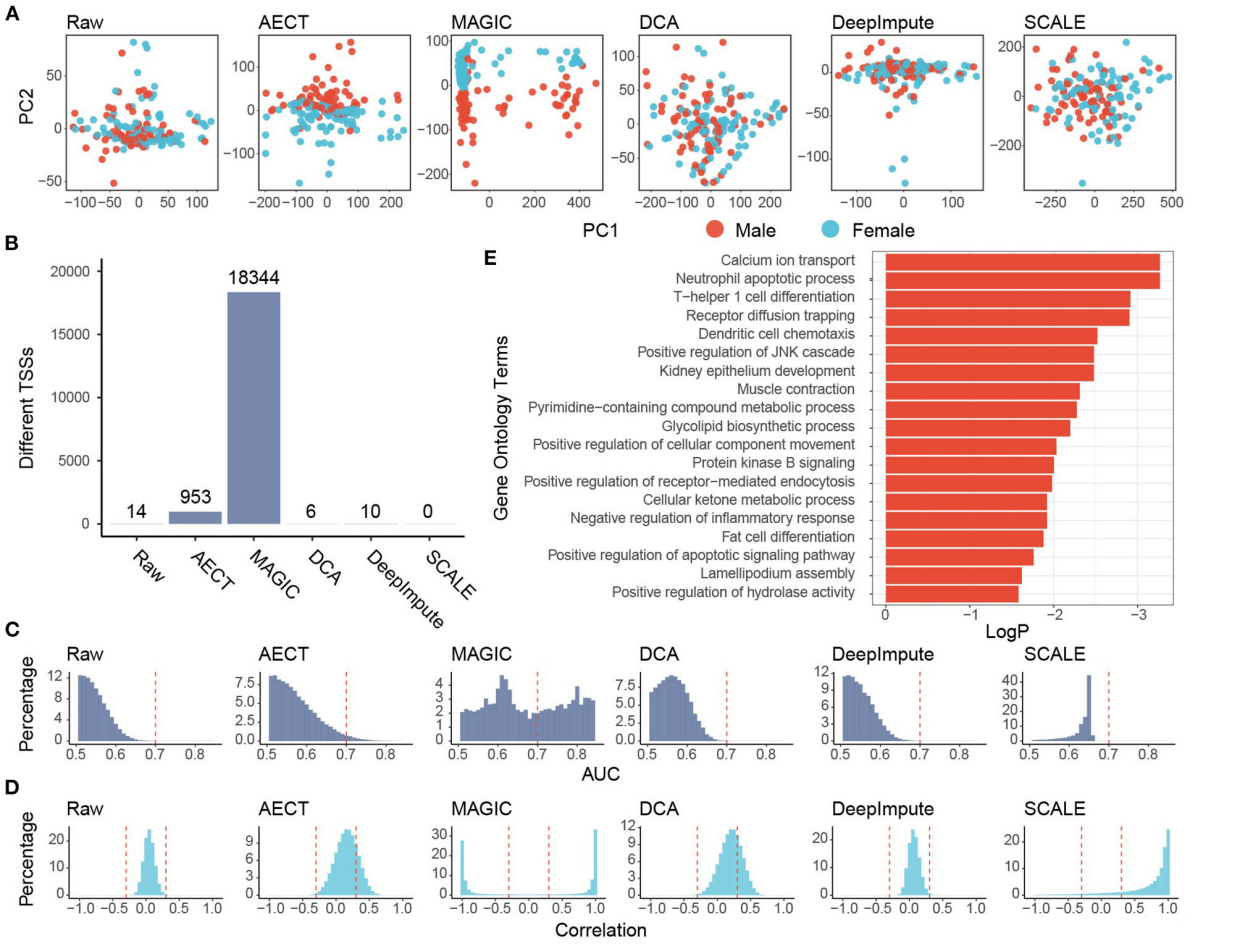
Imputation performance of AECT and other algorithms on healthy donors. **(A)** Plots of principal components 1 and 2 derived from raw TSS coverage profiles and TSS coverage profiles imputed by different algorithms. **(B)** Sex-different TSSs (FDR < 0.1, Wilcoxon rank-sum test adjusted by Holm procedure) identified with different algorithms. **(C)** AUROC patterns of each TSS on sex discrimination; the dashed lines represent AUROC = 0.7. **(D)** Correlation patterns between TSSs; the dashed lines represent Pearson correlation coefficient *r* = ±0.3. **(E)** Gene Oncology enrichment of sex-different TSSs identified by AECT.

To test whether AECT captured common features in the real data, we sequenced the cfDNA of 159 healthy donors with an average of 8.6 M reads (range 7.2 to 10.6 M). PCA was performed on GC-adjusted TSS profiles, and samples were into two groups by donor sex to reflect the differences in TSS profiles between males and females (Supplementary Figure 5). However, when performing PCA with TSSs on autosomes, shallow-sequencing samples cannot be well separated based on sex (Figure 3A). As previously reported, although the differences are not as extensive as with the genes located in sex chromosomes, many autosomal genes have sex-differential transcription patterns (23). We used AECT to capture small differences in autosomal TSS profiles, and the latent features of sex difference were extracted using only the TSS profiles of autosomal genes (Figure 3A). Other algorithms were also performed on autosomal TSS profiles, and only MAGIC successfully distinguished between samples of different sexes (Figure 3A).

Some gene-based evaluations were also performed. First, we compared the ability to identify sex-different TSSs of imputation algorithms. When the TSS profiles were used without imputation, only 14 TSSs showed significant sex differences (FDR < 0.1, Wilcox rank-sum test adjusted by Holm procedure, Figure 3B), and AECT identified 953 sex-different TSSs (FDR < 0.1, Wilcoxon rank-sum test adjusted with the Holm procedure, Figure 3B), and these could discriminate sex differences with area under the receiver operating characteristic curve (AUROC) > 0.7, which implies that AECT recovered the differences between the sexes (Figure 3C). Next, we used gene–gene correlation coefficients to analyze the gene relationships recovered in the algorithms. AECT significantly increased the correlation coefficients among genes (*p* < 2.2 × 10^−16^, Wilcoxon rank-sum paired test), which implies a reconstruction of gene–gene relationships (Figure 3D). The MAGIC algorithm tremendously increased the number of sex-differential TSSs and the gene–gene correlation levels. However, approximately half of TSSs were identified as sex-different, and most gene pairs presented high correlations (Pearson correlation coefficient |*r*| > 0.8), suggesting an over-adjustment in the TSS profiles (Figures 3C,D). Thus, AECT is the only model to perform well on these shallow TSS profiles.

We further investigated whether the TSS profiles imputed by AECT reflect the sex-different biology, and genes with sex-different TSSs identified by AECT were selected for functional enrichment (Figure 3E). Because the cfDNA mostly originated from peripheral blood leucocytes, many enriched Gene Ontology (GO) terms were associated with the biological process of leucocytes. Moreover, similar to the sex-differential transcriptome reported previously (23), GO terms associated with calcium ion transport, muscle contraction, lipid biosynthetic process, ketone metabolism, and fat cell differentiation were significantly enriched (Figure 3E), suggesting that our AECT algorithm recovered the biological status of sex differences.

### AECT Captures the Molecular Characteristics of Breast Cancer

**Figure 4:**
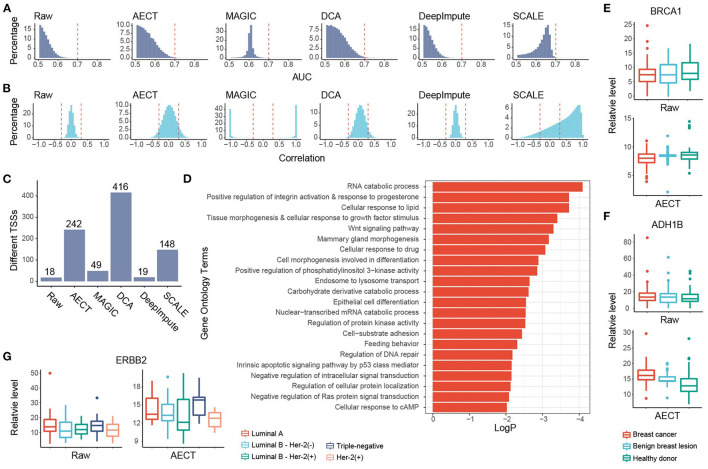
Imputation performance of AECT and other algorithms on breast cancer. **(A)** AUROC patterns for each TSS in breast cancer detection; dashed lines represent AUROC = 0.7. **(B)** Correlation patterns between TSSs; dashed lines represent Pearson correlation coefficients *r* = ±0.3. **(C)** Breast cancer-associated TSSs (FDR < 0.1, Wilcoxon rank-sum test adjusted by Holm procedure) identified with different algorithms. **(D)** Gene Oncology enrichment of sex-different TSSs identified by AECT. **(E,F)** TSS coverage of **(E)** *BRCA1* and **(F)** *ADH1B* in breast cancer, benign breast lesion, and healthy donor group (significant differences between breast cancer and non-cancer samples, *BRCA1* raw: *p* = 0.461; *BRCA1* imputed by AECT, *p* = 0.0480; *ADH1B* raw: *p* = 0.327; *ADH1B* imputed by AECT, *p* = 3.23 × 10^−6^; Wilcoxon rank-sum test). **(G)** TSS coverage of *ERBB2* in different breast cancer subtypes (for *ERBB2* raw and *ERBB2* imputed by AECT, *p* = 0.0472, Kruskal–Wallis test; *p* = 0.0129, Wilcoxon rank-sum test between samples with and without Her-2 expression, respectively).

To further investigate whether AECT captures pathological features, we collected plasma from 90 breast cancer patients and 70 benign breast lesion patients, and ~8.5 M reads of cfDNA sequencing data were generated per sample (range 7.0 to 10.5 M). Another 45 healthy women donors were also included in the analyses (Supplementary Table 1). To reduce the effects of copy number variation in tumor samples, TSS coverage was normalized by relative copy number for all samples. AECT and the four single-cell algorithms were used to impute the TSS coverage profiles. Unfortunately, none of the algorithms could separate cancer patients and non-cancer donors with PCA alone (Supplementary Figure 6), which might be due to the high heterogeneity of the tumors. Alternatively, to evaluate whether the imputation algorithms could capture the differences between breast cancer and non-cancer samples, we calculated the AUROC of each TSS for cancer detection. An ideal distribution curve for an AUROC should have a peak near 0.5 and decrease with increased AUROC because most genes are not relevant to breast cancer. AECT significantly increased the AUROC patterns relative to the raw data without changing the distribution mode (*p* < 2.2 × 10^−16^, Wilcoxon rank-sum paired test) and increased the detection numbers of breast cancer-associated TSSs with AUROC > 0.7 (Figure 4A), while most other algorithms did not produce appropriate AUROC distributions. Meanwhile, using random permutations, we found that AECT did not increase the median AUROC levels of randomly assumed sample types (*p* = 0.140, Wilcoxon rank-sum test, Supplementary Figure 7), suggesting that it captured the particular differences between breast cancer patients and non-cancer donors. Gene–gene correlations were also analyzed in the imputed breast cancer dataset. As previously with purely healthy donors, AECT reconstructed the gene–gene relationships with significantly increased correlation coefficients (*p* < 2.2 × 10^−16^, Wilcoxon rank-sum paired test, Figure 4B).

GO enrichment was performed using 242 breast cancer-associated TSSs identified by AECT (FDR < 0.1, Wilcoxon rank-sum test adjusted by Holm procedure, Figure 4C). A set of GO terms associated with breast cancer were enriched, such as RNA catabolic process, response to progesterone, cellular response to growth factor stimulus, Wnt signaling pathway, and others (Figure 4D), suggesting that AECT recovered the biology of breast cancer. AECT also recovered TSS-coverage levels of a single gene. Although the transcription levels were not always associated with the chromatin status of TSS, a set of typical markers of breast cancer showed a change in TSS coverage after imputation (24). For example, *BRCA1*, which did not change significantly between breast cancer and non-cancer samples in the raw data (*p* = 0.461, Wilcoxon rank-sum test), showed significantly lower TSS coverage in breast cancer patients after imputation (*p* = 0.0480, Wilcoxon rank-sum test), which is consistent with the high expression level of *BRCA1* in breast cancer tissues (Figure 4E). Another example is *ADH1B*, one of the most downregulated genes in breast cancer samples in TCGA (24), which showed significantly higher TSS coverage after AECT imputation (*p* = 3.23 × 10^−6^, Wilcoxon rank-sum test, Figure 4F). Moreover, AECT also contributed to differentiating breast cancer subtypes. After AECT imputation, the marker gene Her2 (*ERBB2*) showed lower TSS coverage in Her-2(+) subtype and luminal B subtype with Her-2 expression (*p* = 0.0472, Kruskal–Wallis test, Figure 4G). These results suggest that AECT-imputed data showed better agreement with the biology of breast cancer, which may be obscured by the shallow sequencing.

### AECT Reflects Molecular Characteristics in Rectal Cancer

**Figure 5:**
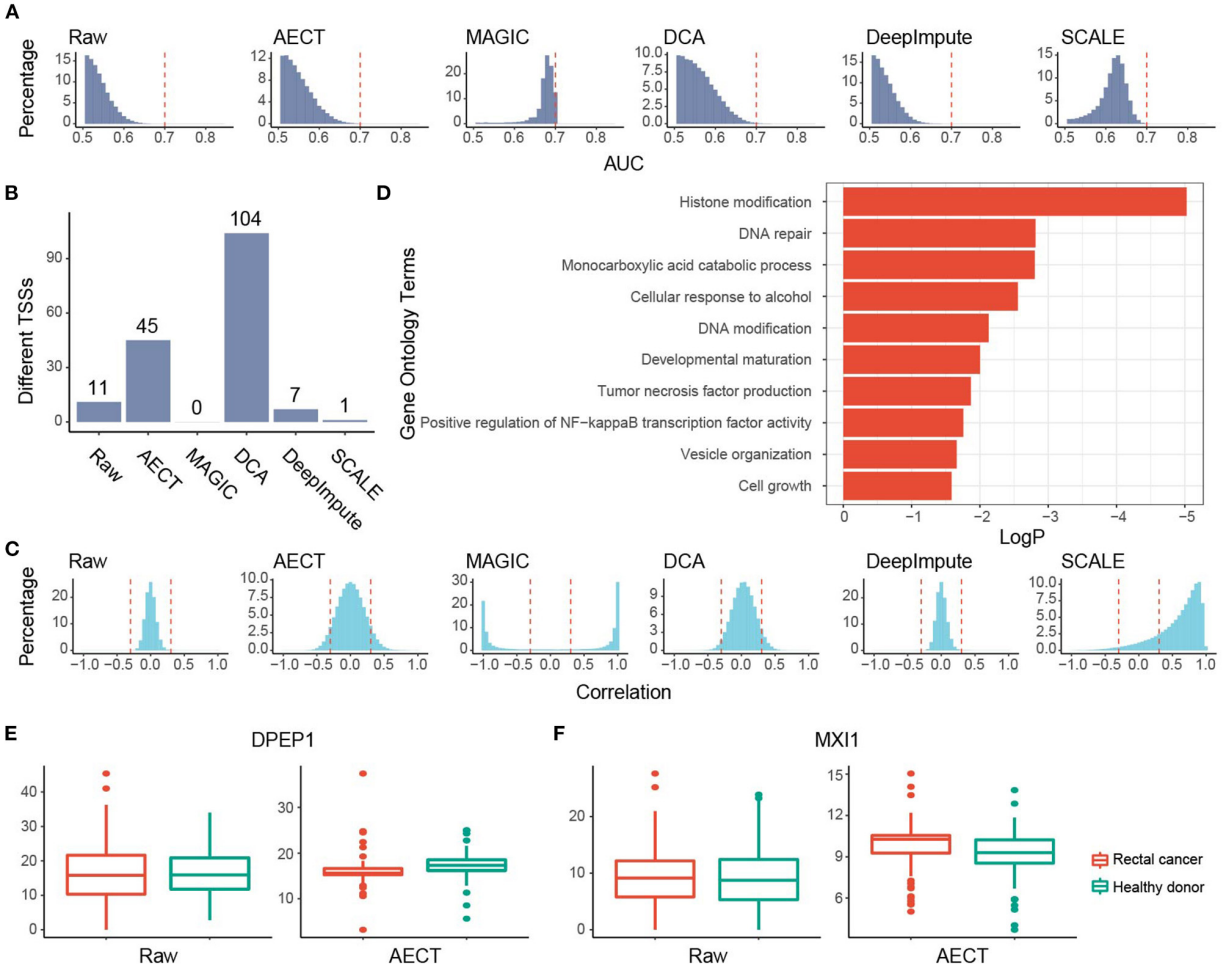
Imputation performance of AECT and other algorithms on rectal cancer. **(A)** AUROC patterns for each TSS on rectal cancer detection; dashed lines represent AUROC = 0.7. **(B)** Rectal cancer-associated TSSs (FDR < 0.1, Wilcoxon rank-sum test adjusted by Holm procedure) identified by different algorithms. **(C)** Correlation patterns between TSSs; dashed lines represent Pearson correlation coefficients *r* = ±0.3. **(D)** Gene Oncology enrichment of sex-different TSSs identified by AECT. **(E,F)** TSS coverage of **(E)** *DPEP1* and **(F)** *MXI1* in breast cancer, benign breast lesion, and healthy donor groups (*DPEP1* raw: *p* = 0.983; *DPEP1* imputed by AECT, *p* = 5.75 × 10^−4^; *MXI1* raw: *p* = 0.730; *MXI1* imputed by AECT, *p* = 0.00146; Wilcoxon rank-sum test).

We also examined AECT's ability to uncover features of another cancer type, namely, rectal cancer. Plasma cfDNA sequencing data of 90 rectal cancer patients were collected and imputed together with 90 healthy donors. Similar to breast cancer patients, whether before or after imputation, PCA could not separate rectal cancer patients from healthy donors (Supplementary Figure 8). AECT increased the AUROC of single genes (*p* < 2.2 × 10^−16^, Wilcoxon rank-sum paired test) and identified more altered TSSs (Figures 5A,B; Supplementary Figure 9), suggesting significantly increased differences between samples from rectal cancer patients and healthy donors. Additionally, AECT also reconstructed gene–gene relationships in rectal cancer datasets (*p* < 2.2 × 10^−16^, Wilcoxon rank-sum paired test, Figure 5C). GO enrichment was performed on most altered 200 TSSs between rectal cancer patients and healthy donors, and GO terms associated with cancer, including histone modification, DNA repair, DNA modification, and tumor necrosis factor production, were enriched (Figure 5D). Typical differentially expressed genes, such as *DPEP1* and *MXI1* (24), also showed altered TSS coverage patterns after AECT imputation, suggesting that AECT also performed well on rectal cancer (Figures 5E,F).

### AECT Improves the Accuracy of Cancer Detection

**Figure 6:**
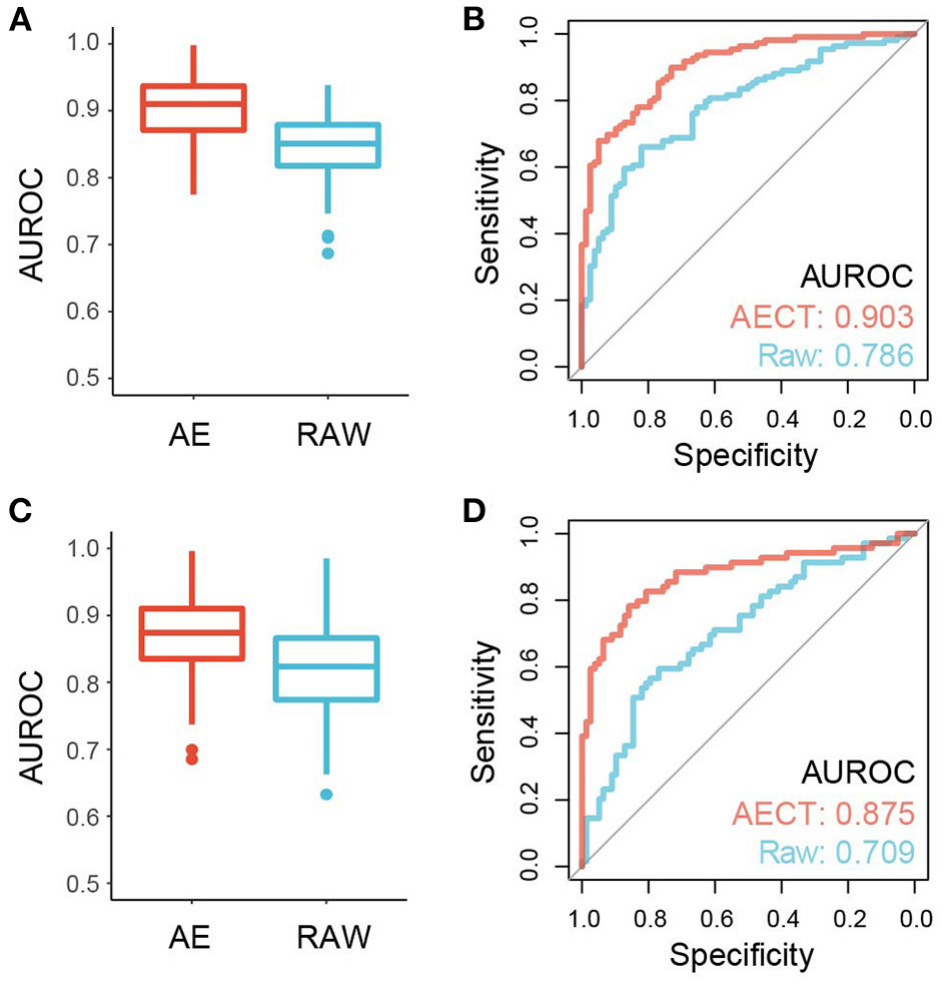
Performance of cancer detection using AECT-imputed data. **(A)** Median AUROC of 100-times permutation with 5-fold cross validation on a training cohort of breast cancer datasets (*p* = 4.62 × 10^−13^, Wilcoxon rank sum test). **(B)** ROC curve of validation cohort of breast cancer datasets (*p* = 7.36 × 10^−4^, Delong test). **(C)** Median AUROC of 100-times permutation with 5-fold cross validation on a training cohort of rectal cancer datasets (*p* = 3.35 × 10^−8^, Wilcoxon rank sum test). **(D)** ROC curve of validation cohort of rectal cancer datasets (*p* = 1.78 × 10^−3^, Delong test).

TSS coverage profiles are widely used to detect cancer or other pathological states, but their performance is barely satisfactory due to low sequencing depths (3, 10, 11). Because AECT increased the AUROC of single TSSs and improved the quantification of TSS coverage levels, we speculated that AECT-imputed data may detect cancer more precisely. A bootstrapping-based LASSO algorithm was employed to build classifiers for breast cancer patients and non-cancer donors, and an independent validation cohort was used for performance evaluation (Supplementary Table 1). Using raw TSS profiles without imputation, our model produced similar accuracy to that reported in previous studies (3, 10, 11) (median AUROC of 5-fold cross validation = 0.847 in training cohort, AUROC = 0.786 in validation cohorts, Figures 6A,B). As expected, AECT-imputed data significantly increased detection accuracy (median AUROC of 5-fold cross validation = 0.909 in training cohort, Wilcoxon rank sum test *p* = 4.62 × 10^−13^; AUROC = 0.903 in validation cohorts, Delong test *p* = 7.36 × 10^−4^, Figures 6A,B). Similar analyses were also performed on rectal cancer datasets, and it was found that AECT improved detection performance in them as well (training cohort: median AUROC of 5-fold cross validation = 0.823 and 0.876 for raw data and imputed data, respectively, Wilcoxon rank sum test *p* = 3.35 × 10^−8^, Figure 6C; validation cohort: AUROC = 0.709 and 0.875 for raw data and imputed data, respectively, Delong test *p* = 1.78 × 10^−3^, Figure 6D).

## Discussion

TSS coverage profiles have been widely shown to reflect physiological and pathological conditions; however, accurate quantification of them requires high-depth sequencing data, which is hardly been satisfied in clinical applications (3, 5–8, 10). To deal with shallow sequencing data, we previously merged TSS with similar coverage trends among groups; unfortunately, this method was insufficient for disease prediction and could not provide precise coverage for each TSS (11). Hence, we introduced AECT, which is tailored to TSS coverage matrices generated by shallow sequencing data. In this study, we found that it could impute TSS coverage profiles using low-coverage data without loss of latent biological features. We also compared AECT with representative algorithms designed for single-cell sequencing data, including MAGIC (12), DeepImpute (15), DCA (13), and SCALE (14). Although DCA and DeepImupte, generated high correlation and low MSE in simulated low-coverage data, AECT is the only algorithm which separated samples with different sex. Moreover, AECT captures the molecular characteristics of cancer patients, thus AECT had higher overall accuracy than imputation algorithms designed for single-cell RNA-seq or single-cell ATAC-seq data in both simulated and experimental datasets.

It is worth noting that the evaluation of imputation is difficult for real datasets because there are few available cfDNA datasets with sufficient depth for extremely precise TSS coverage quantification. However, trends in RNA expression and TSS openness are not always consistent (25), so RNA-seq data are not suitable for evaluation. Nevertheless, we performed a set of indirect analyses to evaluate the performance of AECT. AECT showed benefits in specifically increasing overall differences between different biological statuses and identifying significantly changed TSSs, suggesting that it captured internal features in the samples. AECT also increased gene–gene correlations in shallow sequencing data, which could contribute to establishing regulatory networks using cfDNA. Because plasma cfDNA is primarily derived from apoptotic immunocytes (6), our algorithm could provide a method for understanding the biology that underlies immunologic processes for both healthy individuals and tumor patients (26, 27). Thus, AECT may lead to a set of applications with lower cost in different fields. Early detection, differential diagnosis, and companion diagnostics of cancer might be the biggest potential applications, because AECT could reflect physiological conditions with an acceptable price. Similar applications are also suitable for immunological diseases, because cfDNA is mainly derived from immune cells. Monitoring of the physiology and pathology of pregnancy might be another field for AECT, considering NIPT has been widely used, there may be no additional cost for prediction of physiological and pathological prediction.

An additional advantage of AECT is the improvement of classifier performance, which might because of the enhanced robustness of TSS coverage quantification. Using shallow sequencing data, AECT achieved acceptable detection accuracy, close to the results achieved using high-depth data (7, 28). Considering that the clinical application of TSS profiling is limited by its high cost, AECT could significantly reduce the budget for high-throughput sequencing, which may enable moderate cost platforms for health monitoring, cancer screening, prediction of pregnancy complications, and other clinical usages.

Several optimizations may further improve the performance of the imputation algorithms. For example, AECT uses the MSE loss function, but the TSS coverage profiles are not strictly normally distributed. Thus, an autoencoder fit for the distribution likelihood (13) or fit for the multimodal distribution (14) may further improve the imputation performance. Generally, the sample sizes for cfDNA sequencing data are not as large as those in single-cell sequencing, so fitting methods may lead to overfitting and over-imputation of the data. For this reason, regularization methods such as neuron dropout, L1 regularization, and L2 regularization should be introduced into the model. On the other hand, because plasma cfDNA is derived from different tissues, the imputation performance may benefit from incorporating additional biological variables, such as cfDNA fetal fraction for NIPT samples and cfDNA tumor fraction for tumor samples. We have made AECT an available Python package and Docker file on GitHub: https://github.com/hanbw0120/AECT.

## Conclusion

We developed a deep-learning pipeline, namely AECT, for TSS coverage profiles generated from cfDNA sequences. Outperforming existing single-cell sequencing imputation algorithms, AECT reflects molecular characteristics in healthy donors and cancer patients, and classifiers show that using AECT works well on cancer detection.

## Data Availability Statement

Publicly available datasets were analyzed in this study. This data can be found here: SRA (https://www.ncbi.nlm.nih.gov/bioproject/PRJNA683983, project ID: PRJNA683983) and NODE (https://www.biosino.org/node, project ID: OEP000648).

## Author Contributions

X-XY, S-FQ, and Y-SW designed the study. XY, L-MH, and KL performed the experiments. B-WH, Z-WG, G-JO, SX, and R-TW analyzed data. G-XC and W-WX provided clinical information. B-WH, XY, X-XY, and Y-SW wrote the manuscript. All authors revised the manuscript. All authors contributed to the article and approved the submitted version.

## Funding

The work was supported by National Natural Science Foundation of China (81900191), Medical Scientific Research Foundation of Guangdong Province of China (B2017006), and China Postdoctoral Science Foundation funded project (2019M662998).

## Conflict of Interest

KL was employed by the company Guangzhou XGene Co., Ltd., and G-JO, R-TW, and SX were employed by the company Guangzhou Darui Biotechnology Co., Ltd. The remaining authors declare that the research was conducted in the absence of any commercial or financial relationships that could be construed as a potential conflict of interest.

## Publisher's Note

All claims expressed in this article are solely those of the authors and do not necessarily represent those of their affiliated organizations, or those of the publisher, the editors and the reviewers. Any product that may be evaluated in this article, or claim that may be made by its manufacturer, is not guaranteed or endorsed by the publisher.
