# Supplementary material for: A Deep-Learning Pipeline for TSS Coverage Imputation From Shallow Cell-Free DNA Sequencing
Source: Front Med (Lausanne). 2021 Dec 3;8:684238. doi: 10.3389/fmed.2021.684238 (PMC8678047; doi:10.3389/fmed.2021.684238)
Supplement: Supplementary file 1 [file Table_1.DOCX]

**Supplementary Table 1. Clinical characteristics**

|  | Health Donors (159) | Breast cancer (168) | Benign breast lesion (140) | Rectal cancer (168) |
| --- | --- | --- | --- | --- |
| **Age (median and range)** | | | | |
|  | 32 (21-42) | 47 (26-68) | 44 (20-74) | 57.5 (24-78) |
| **Sex** |  |  |  |  |
| Male | 74 (46.5%) | 0 (0.0%) | 0 (0.0%) | 102 (60.7%) |
| Female | 85 (53.5%) | 168 (100.0%) | 140 (100.0%) | 66 (39.3%) |
| **Stage** |  |  |  |  |
| I | - | 26 (15.5%) | - | 0 (0.0%) |
| II | - | 93 (55.4%) | - | 28 (16.7%) |
| III | - | 47 (28.0%) | - | 140 (83.3%) |
| IV | - | 2 (1.2%) | - | 0 (0.0%) |
| **Subtype** |  |  |  |  |
| Luminal A | - | 36 (24.1%) | - | - |
| Luminal B | - | 77 (45.8%) | - | - |
| Her-2 | - | 24 (14.3%) | - | - |
| TNBC | - | 23 (13.7%) | - | - |
| Unclassified | - | 8 (4.8%) | - | - |
