# Supplementary material for: A Deep-Learning Pipeline for TSS Coverage Imputation From Shallow Cell-Free DNA Sequencing
Source: Front Med (Lausanne). 2021 Dec 3;8:684238. doi: 10.3389/fmed.2021.684238 (PMC8678047; doi:10.3389/fmed.2021.684238)
Supplement: Supplementary file 3 [file Data_Sheet_1.PDF]

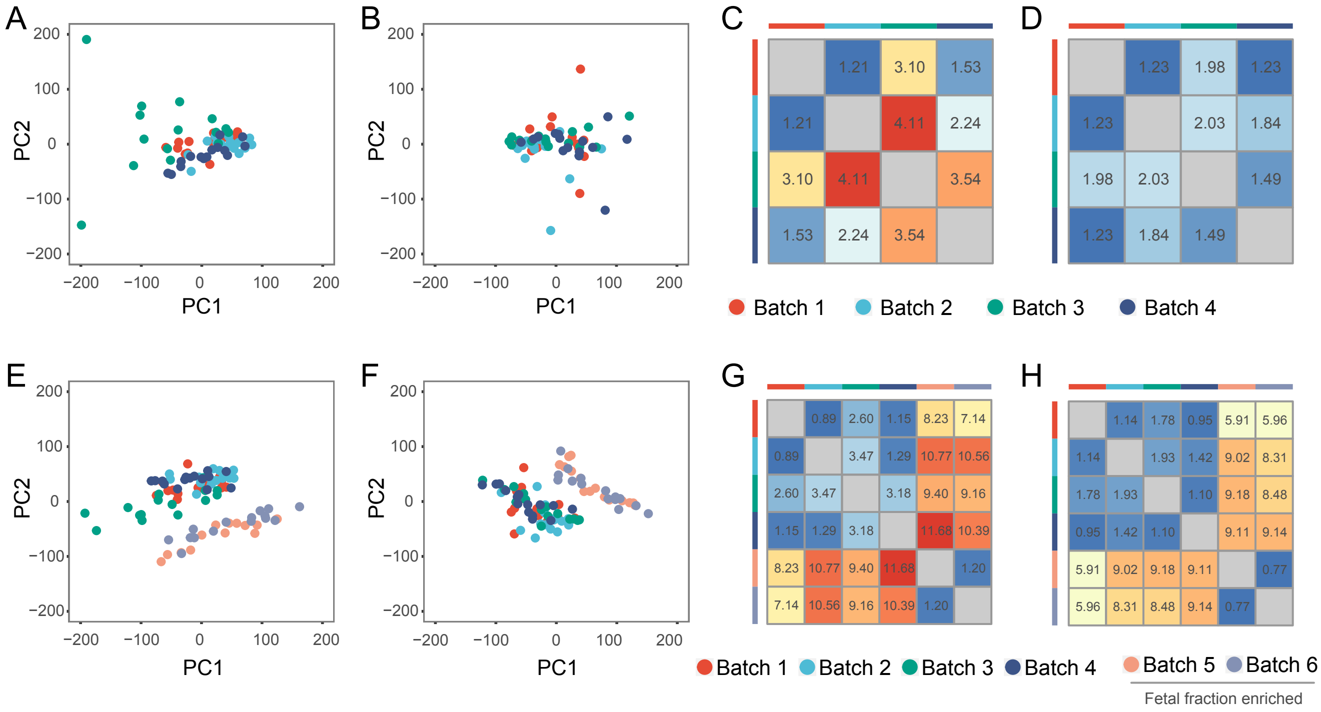


Figure S1. PCA plots of TSS coverage profiles with and without GC bias adjustment.
A, B. TSS coverage profiles with (A) and without (B) GC bias adjustment generated from standard NIPT data of 5 batches.
C, D. Bhattacharyya distance matrix of different NIPT batches, calculated using TSS coverage profiles with (C) and without (D) GC bias adjustment.
E-F. TSS coverage profiles with (E) and without (F) GC bias adjustment generated from 5 batches standard NIPT data and 2 batches fetal fraction enrichment NIPT data.
G-H. Bhattacharyya distance matrix of different NIPT batches, calculated using TSS coverage profiles with (G) and without (H) GC bias adjustment.

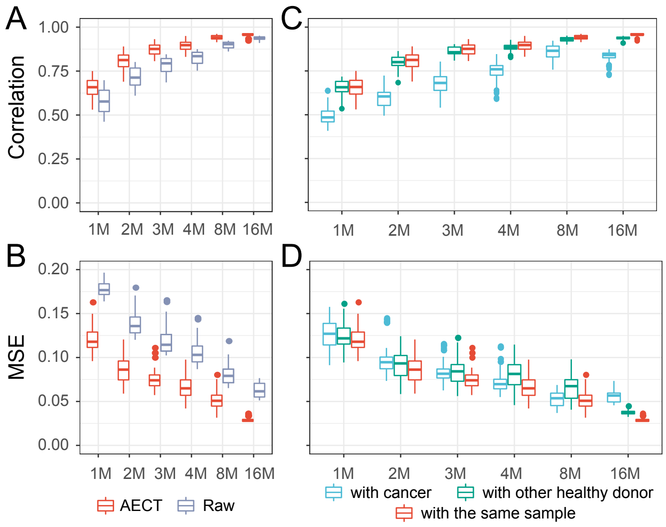


Figure S2. AECT improved the accuracy of TSS coverage profiles in stimulated data from healthy donors.

A, B. AECT increased Pearson correlation coefficient (A) and decreased MSE (B) of shallow data and original high depth data.

C, D. AECT generated higher Pearson correlation coefficients (C) and lower MSEs (D) with high-depth data from healthy donors than with high-depth data from cancer samples. The x-axis represents the read counts of each stimulated shallow sequencing data. Boxes represent interquartile ranges, horizontal lines in the box are the medians, and the whiskers show 1.5 times the interquartile ranges.

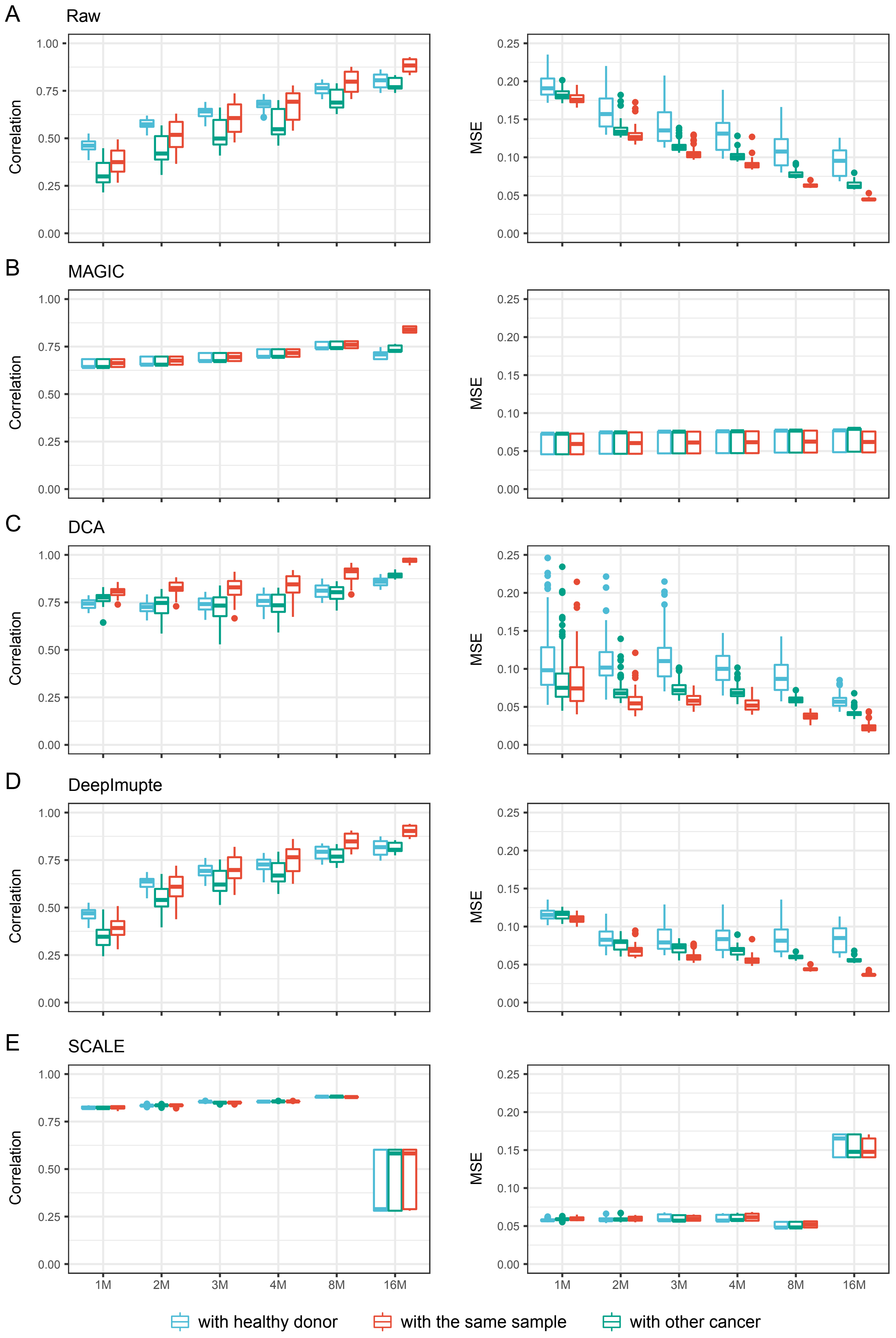


Figure S3. Performance of other imputation algorithms in stimulated data for cancer patients. The Pearson correlation coefficients and MSEs of the original high-depth data and shallow data were generated by (A) raw data, (B) MAGIC, (C) DCA, (D) DeepImpute, and (E) SCALE.

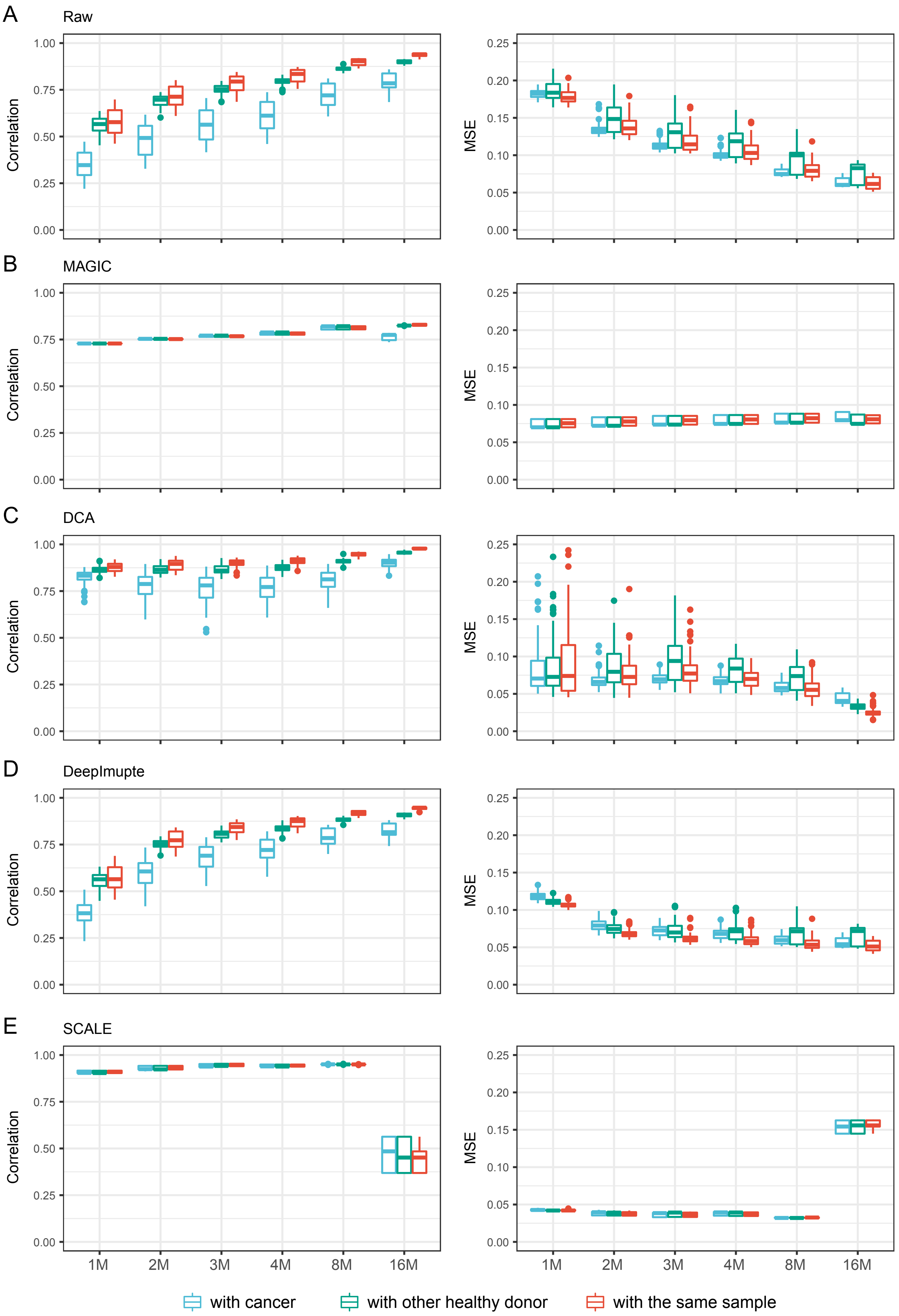


Figure S4. Performance of other imputation algorithms in stimulated data for healthy donors. The Pearson correlation coefficients and MSEs of original high-depth data and shallow data were generated by (A) raw data, (B) MAGIC, (C) DCA, (D) DeepImpute, and (E) SCALE.

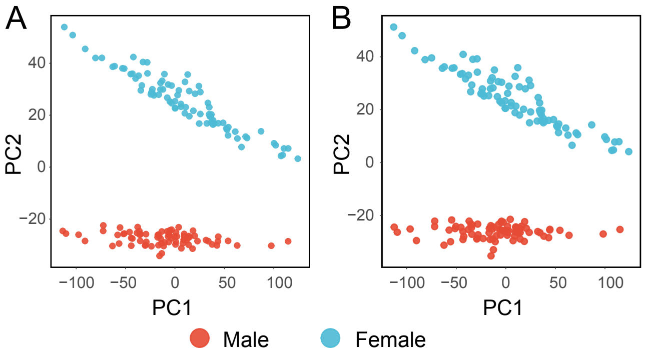


Figure S5. PCA plot derived from raw TSS coverage profiles of healthy donors. TSSs from all chromosomes (A) and all chromosomes except for chrY (B) were subjected to analyses.

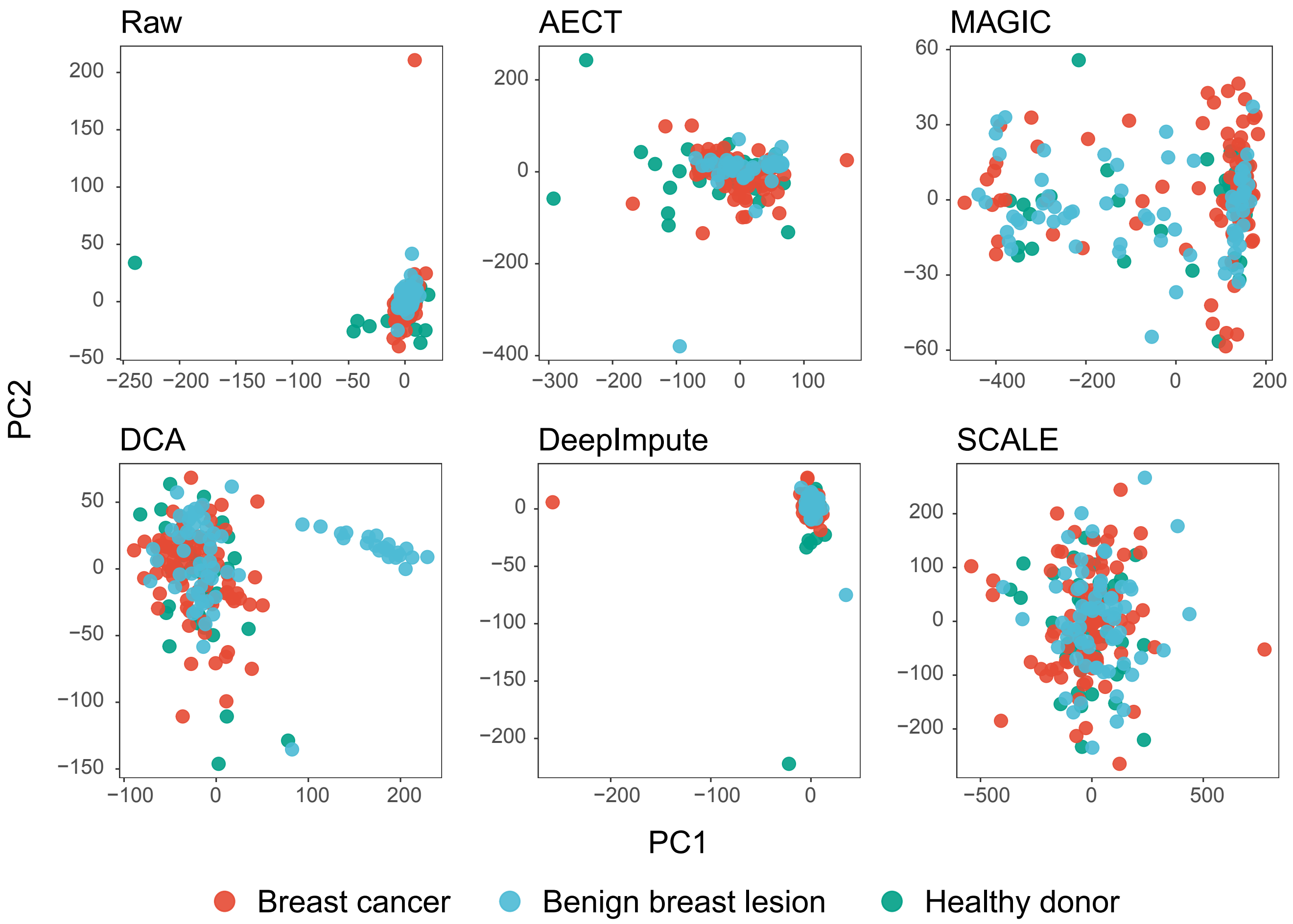


Figure S6. PCA plot derived TSS coverage profiles imputed by different algorithms for breast cancer datasets.

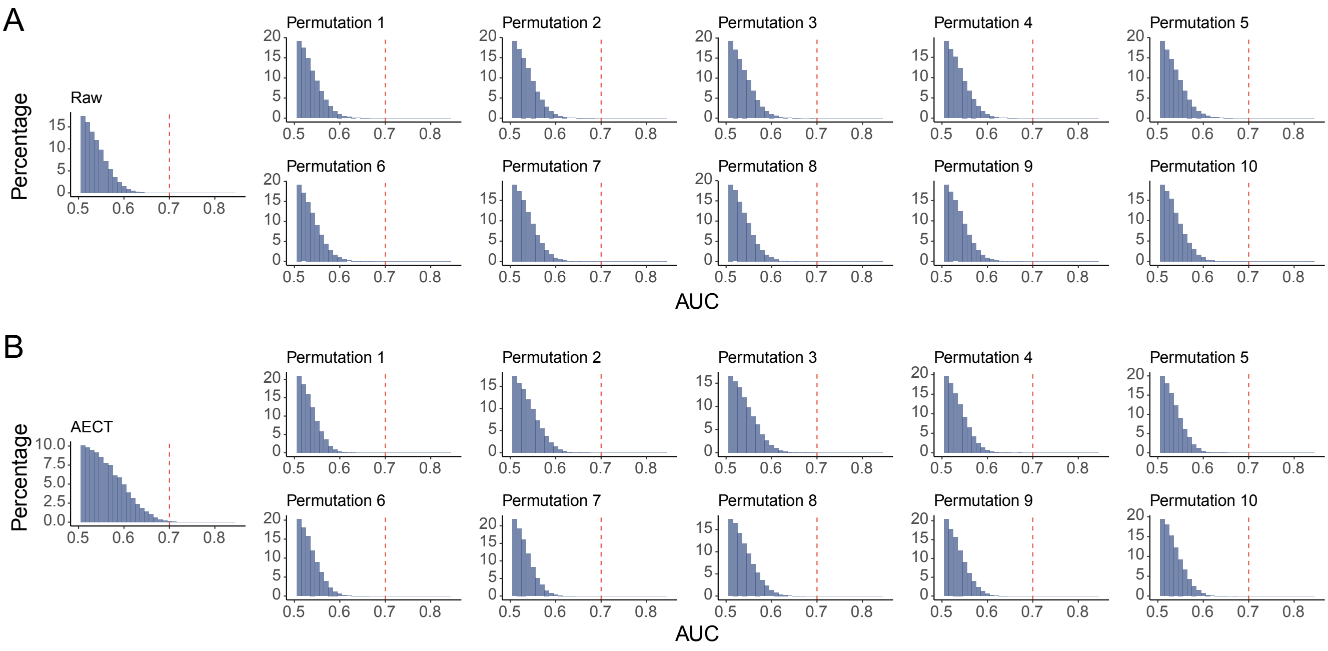


Figure S7. AUROC patterns for each TSS in breast cancer detection in (A) raw data and (B) AECT-imputed data. The left panel shows the original patterns, and the right panel was generated through 10-times randomly assumed sample types. In the random permutations, there was no significant difference between the median AUROC with and without imputation ($p = 0.140$, Wilcoxon paired rank sum test).

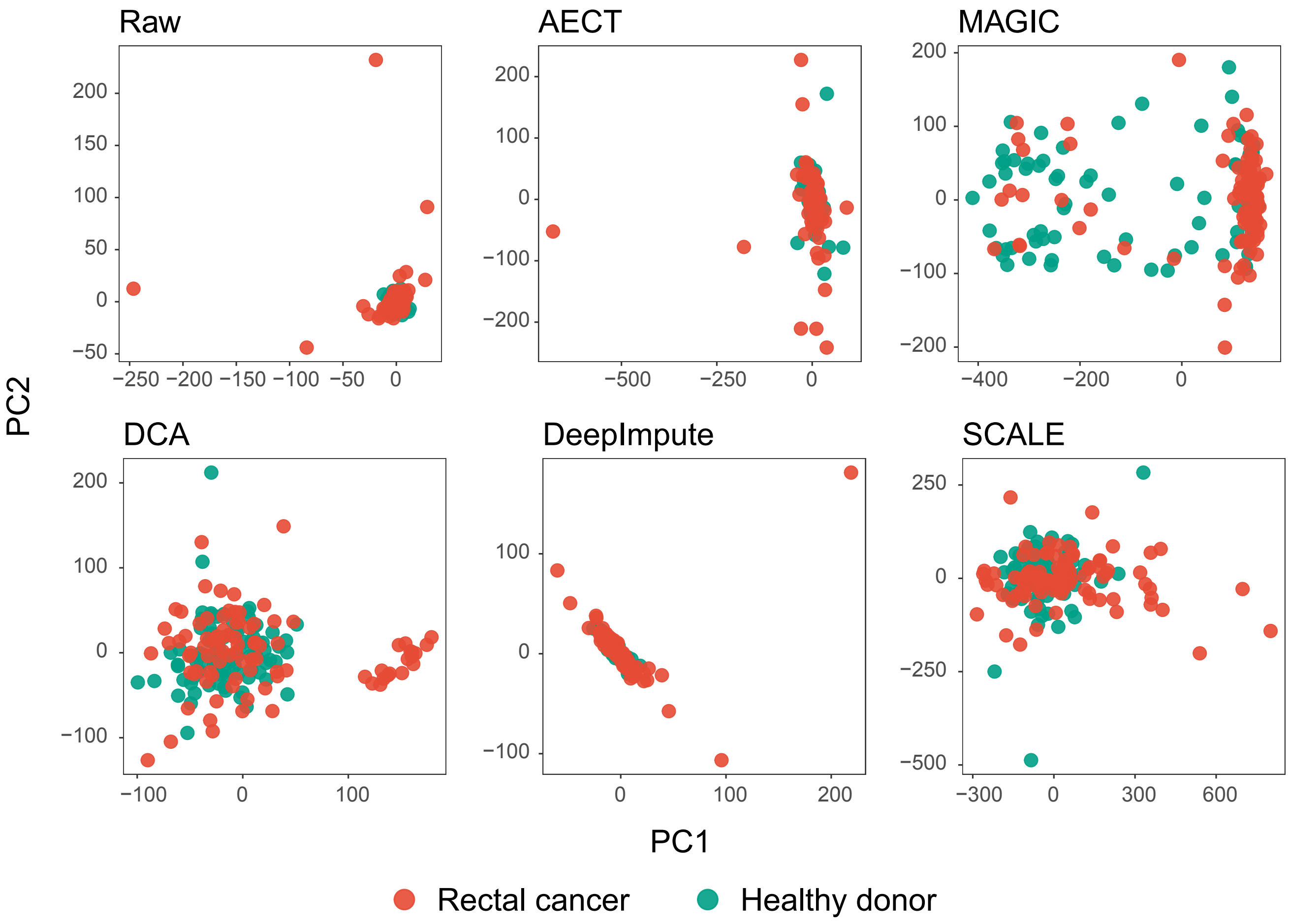


Figure S8. PCA plot derived TSS coverage profiles imputed with different algorithms for rectal cancer datasets.

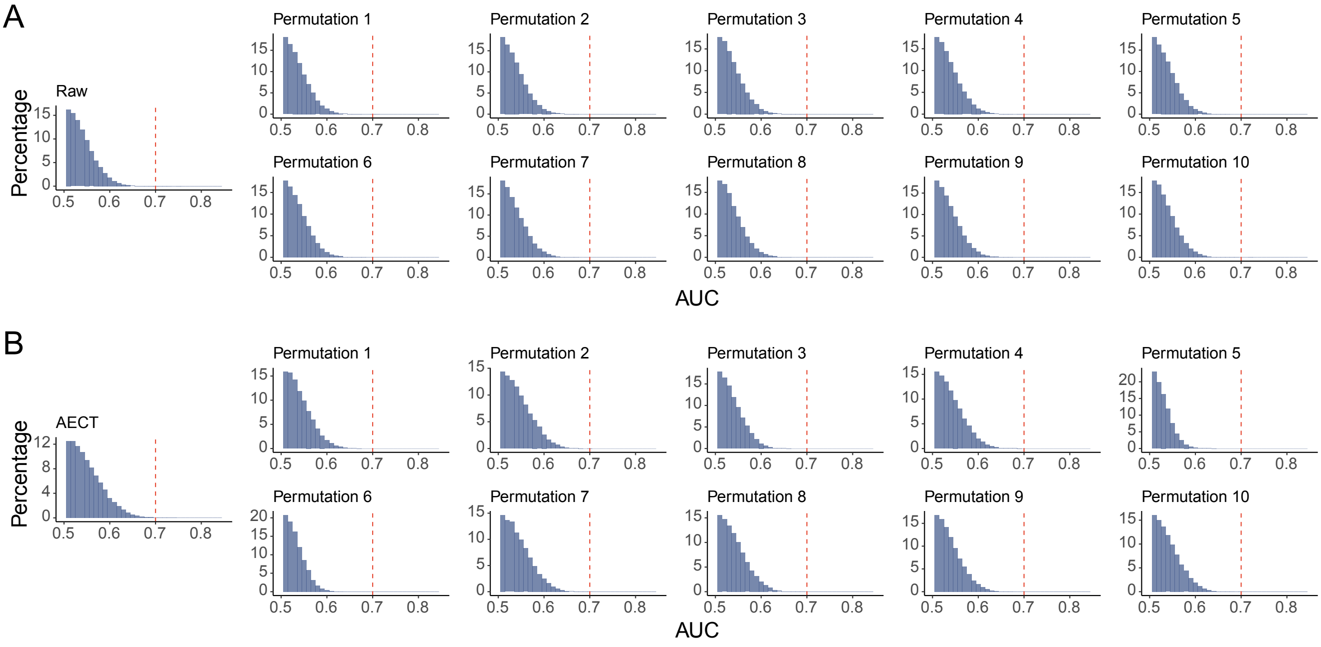


Figure S9. AUROC patterns for each TSS in the rectal cancer detection of (A) raw data and (B) AECT-imputed data. The left panel shows the original patterns, and the right panel was generated using 10-times randomly assumed sample types. In the random permutations, there was no significant difference between the median AUROC with and without imputation (p = 0.290, Wilcoxon paired rank sum test).
